# Evidence of dengue virus transmission and a diverse *Aedes* mosquito virome on the Democratic Republic of Congo-Angola border

**DOI:** 10.1101/2025.01.16.633031

**Published:** 2025-09-23

**Authors:** Wenqiao He, Thierry Bobanga, Anne Piantadosi, Zachary R. Popkin-Hall, Fabien Vulu, Matthew H. Collins, Melchior M. Kashamuka, Antoinette K. Tshefu, Jonathan J. Juliano, Jonathan B. Parr

**Affiliations:** 1 Institute for Global Health and Infectious Diseases and Division of Infectious Diseases, Department of Medicine, University of North Carolina at Chapel Hill, Chapel Hill, NC, United States; 2 Department of Tropical Medicine, Faculty of Medicine, University of Kinshasa, Kinshasa, Democratic Republic of the Congo; 3 Department of Pathology and Laboratory Medicine, Emory University School of Medicine, Atlanta, GA, USA; 4 Division of Infectious Diseases, Department of Medicine, Emory University School of Medicine, Atlanta, GA, USA; 5 Kinshasa School of Public Health, Kinshasa, Democratic Republic of the Congo

**Keywords:** *Aedes* mosquito, dengue, Democratic Republic of Congo, viral metagenomic

## Abstract

*Aedes* mosquitoes are widely distributed across the Democratic Republic of Congo (DRC), and are major vectors of dengue (DENV), Zika, chikungunya (CHIKV), and yellow fever (YFV) viruses. While the high burden of malaria in the DRC receives considerable attention, arboviruses remain understudied. In the setting of recent CHIKV and YFV outbreaks in southwestern DRC, we collected *Aedes* mosquitoes in three areas of Kimpese, DRC, near the Angola border, to investigate their virome. Metagenomic and targeted sequencing of eight randomly selected field mosquito pools, comprising 155 mosquitoes from three collection sites, confirmed high-confidence DENV reads and human blood meals in six (75%) and eight (100%) pools, respectively. We find diverse mosquito viromes including other known and putative human and animal viruses. Our findings provide strong evidence of endemic DENV transmission along the DRC-Angola border and illustrate the potential of wild-caught mosquitoes for xenosurveillance of emerging pathogens.

## Introduction

Arthropod-borne pathogens, including viruses, bacteria, and parasites, account for more than 17% of all infectious diseases ^1,2^, and over 700 arboviruses have been documented to infect humans across a wide geographic distribution ^3,4^. The number of outbreaks and endemic infections caused by emergent and re-emergent mosquito-borne arboviruses have increased worldwide over the last two decades ^5–7^. *Aedes* mosquitoes are particularly important vectors for multiple important human pathogens, including dengue virus (DENV), Zika virus (ZIKV), yellow fever virus (YFV), and chikungunya virus (CHIKV) ^8–11^. The warm climate and suitable environmental conditions in tropical regions support the proliferation of both mosquitoes and the viruses they transmit. As a result, tropical regions tend to have higher diversity and prevalence of arboviruses than other regions. However, arbovirus transmission is understudied across Africa, where improved understanding is urgently needed to guide roll out of newly available DENV vaccines, in particular ^12^.

The Democratic Republic of the Congo (DRC) is the largest country in central Africa with a population of over 100 million, and a tropical climate conducive to mosquitoes. Approximately 248 mosquito species are thought to reside in the DRC ^13^, including *Aedes aegypti* and *Aedes albopictus*, both of which are major disease vectors ^7,14^. A recent survey found that *Ae. albopictus* is expanding its distribution in the DRC and displacing native *Aedes* species ^15^. Chikungunya, YFV, and DENV outbreaks and infections are known to occur in the DRC ^16–18^, and increasing reports of mosquito-borne and emerging viral diseases have been recorded ^19^. Considerable attention is devoted to the high burden of malaria ^20^ in the DRC, but arboviruses remain neglected, with limited studies of humans and mosquitoes to-date ^3,17–19,21^. The lack of diagnostic tools and surveillance systems for these diseases hinders our understanding of their impact and epidemiology ^22^.

**Figure 1.**
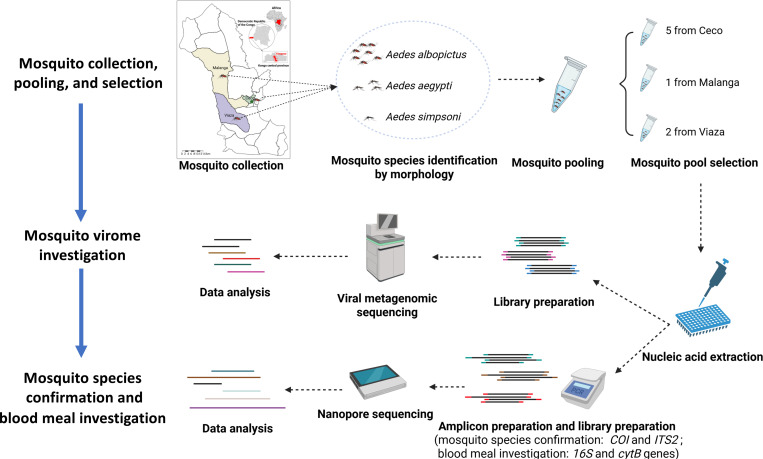
Schematic of the viral metagenomic, mosquito species, and blood meal analysis of *Aedes* mosquitoes from the Democratic Republic of the Congo. Created in BioRender.

Metagenomic sequencing approaches can be used for identification of viral DNA and/or RNA within diverse sample types. These methods have recently been applied to mosquito virome analyses, particularly in the context of mosquito-borne pathogen transmission and xenosurveillance, to enhance understanding of host–vector–environment interactions and support integrated monitoring of pathogens and exposures at the interface of animal and public health ^23–26^. To improve our understanding of the role of *Aedes* mosquito vectors in arboviral transmission in the DRC, we collect and sequence the virome of mosquito pools from three areas of Kimpese, a region near the Angola border which has experienced recent arboviral outbreaks (Figure 1). We observe diverse viromes and identify strong evidence of active DENV transmission in the region, corroborated by viral sequences, DENV PCR, and mosquito blood meal analysis.

## Results

### Mosquito collection and species confirmation

**Figure 2.**
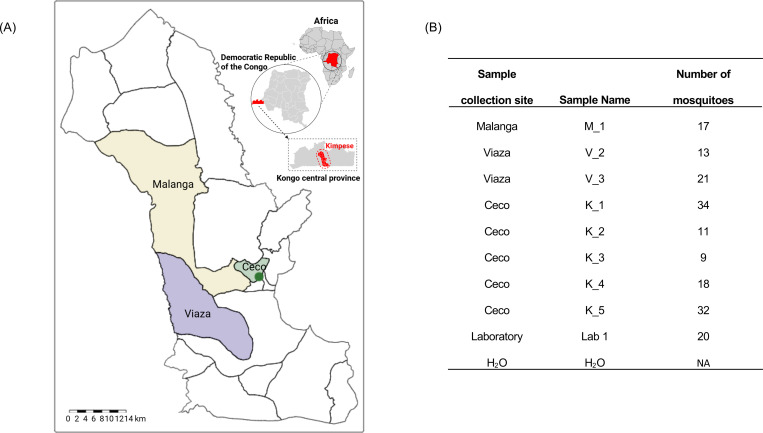
*Aedes* mosquitoes trapped in the DRC. A) Sample collection areas in Kimpese, DRC. Kimpese city is annotated by a green dot. B) Detailed information of the selected mosquito pools, including health areas where they were collected, and including laboratory-reared *Ae. aegypti* (Lab 1) and water (H_2_O) controls.

More than 600 adult *Aedes* mosquitoes (Table S1) were collected and distributed into 37 pools based on sample collection sites (21 pools from Kimpese city, 6 pools from Malanga, and 10 pools from Viaza, Figure 2a). Three species of *Aedes* mosquitoes were identified by morphology: *Aedes albopictus (n=653, 98.34%)*, *Aedes aegypti (n=8, 1.20%)*, and *Aedes simpsoni (n=3, 0.45%)*. Among the eight sequenced pools (Figure 2b), all contained majority *Ae. albopictus* (Table S2) based on their *ITS2* and *COI* gene sequences.

### Sequencing output

**Figure 3.**
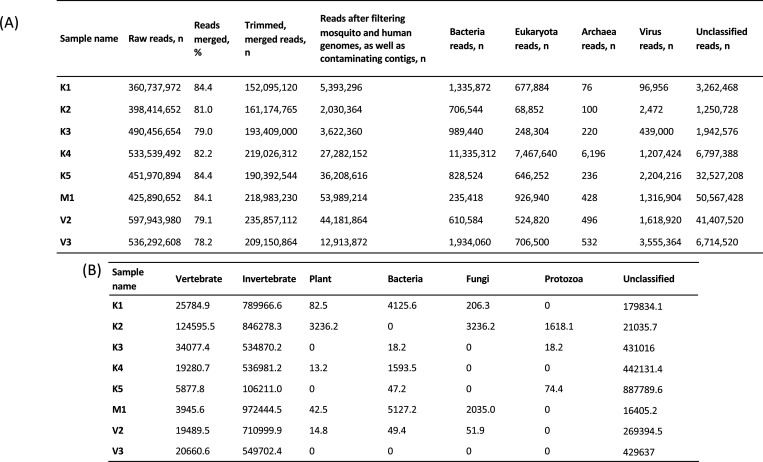
Summary of read counts based on the KrakenUniq nt database. **A)** Summary statistics for sequenced mosquito pools. **B)** Virus reads by presumed host, reported as reads per million based on KrakenUniq annotation using the nt database.

A total of 3,795,246,904 raw reads were obtained by Illumina sequencing. A range of 78.2–84.4% paired-end reads in each field mosquito pool were successfully merged. After filtering out low quality reads and those mapping to host and contaminating contigs (Figure 3a and Table S3), 185,621,738 reads remained, of which 144,469,836 were unclassified reads (77.8% of total) that did not map to any sequences in the KrakenUniq default nt database. Classified reads were mapped to known viruses, bacteria, eukaryota, and archaea.

### Viral metagenomic analysis

Results confirm a highly diverse virome in wild-caught *Aedes* from the DRC, with viruses specific to invertebrates, vertebrates, plants, bacteria, fungi, and protozoa, as well as an unclassified group of viruses (Figure 3b). We do not observe a correlation between the number of viral taxa and the number of mosquitoes in the field-collected mosquito pools.

**Figure 4.**
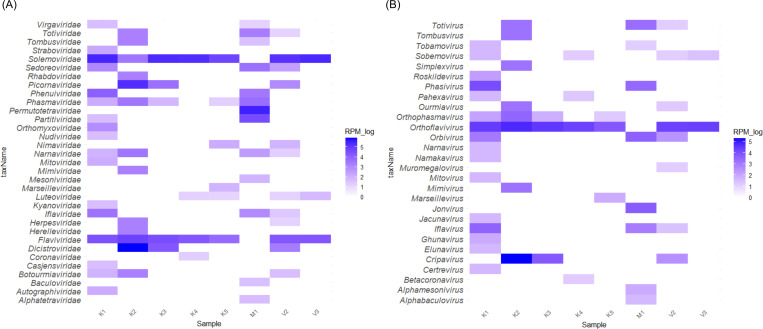
Viral families and genera in field mosquito pools. **A)** Heatmap based on reads per million viral reads in each mosquito pool (family level). **B)** Heatmap based on reads per million viral reads in each mosquito pool (genus level). Per International Committee on Taxonomy of Viruses recommendations, reads of *Orthoflavivirus* comprises both *Orthoflavivirus* and *Flavivirus* identified by KrakenUniq.

Most of the viral genera detected in the laboratory-uninfected *Ae. aegypti* mosquito pool and the water control are not found in the field mosquito pools (Table S4). Although reads annotated as viral genera *Betacoronavirus*, *Mimivirus*, and *Pahexavirus* are detected in both the water control and some field mosquito pools, BLAST results show no true matches to *Betacoronavirus* in the water control and reveal that the *Mimivirus* and *Pahexavirus* reads in field mosquito pools mapped to different genomic regions than those in the water control; therefore, no reads in field mosquito pools are removed in this filtering step. Based on taxonomic classification using the KrakenUniq with the NCBI-nt database, and after applying the RPM < 10 threshold and index-hopping filter, we identify 33 unique total viral families and 28 unique total viral genera in the field mosquito pools, with each pool containing between 3–17 viral families and 2–16 viral genera (Figure 4, S1, and S2, Supplementary Material).

*Flaviviridae* is found in all field mosquito pools collected from Kimpese city and Viaza. *Orthoflavivirus,* which includes viruses formerly classified under the *Orthoflavivirus* and *Flavivirus* genera ^27^, is the most abundant viral genus in most Kimpese mosquito pools (except K2) and all Viaza pools, with a large number of reads mapping to *Orthoflavivirus denguei*. K2 contains a large number of reads annotated as genus *Cripavirus* (family *Dicistroviridae*). *Jonvirus*, a genus typically associated with insect viruses, shows the highest relative abundance in the mosquito pool from Malanga and is exclusively detected in that pool.

Several viral genera capable of infecting humans and animals are detected in mosquito pools collected from Kimpese city. These include *Betacoronavirus* (family *Coronaviridae*), *Marseillevirus* (family *Marseilleviridae*), and *Simplexvirus* (family *Herpesviridae*). Genus *Orthophasmavirus* is found exclusively in Kimpese pools, while *Alphabaculovirus* and *Alphamesonivirus* are detected only in the mosquito pool from Malanga. These genera contain insect-associated viruses. *Muromegalovirus*, a genus within the family *Herpesviridae* known to naturally infect rodents, is detected exclusively in one Viaza pool and is not in mosquito pools from Kimpese city and Malanga. All corresponding reads map to viral species *Muromegalovirus muridbeta2*.

PCA based on detected viral genera to explore clustering by sample collection area is shown in Figure S3 and Table S5. Geographical clustering is observed among samples from Kimpese city and Viaza. Notably, two mosquito pools from Kimpese city differ substantially from the other three pools collected at the same site.

KrakenUniq annotation results prior to applying the RPM < 10 threshold and index-hopping filter are provided in the Supplementary Notes. These datasets include all viral sequences remaining after initial quality filtering steps, such as removal of low-quality reads, host-derived sequences, and contaminant contigs or reads, providing a comprehensive overview of the detected viral diversity. Notably, some pathogens and potential pathogens were detected with low read counts, including *human pegivirus* and *human blood-associated dicistrovirus*.

### Insect-specific viruses

We find a large number of insect-specific viruses in field mosquito pools, with most belonging to the families *Solemoviridae* and *Flaviviridae*. *Wenzhou sobemo-like virus 4*, *Hubei mosquito virus 2*, *Guangzhou sobemo-like virus*, *Sichuan mosquito sobemo-like virus*, and *Aedes flavivirus* are the most prevalent species (Table S6). Except for *Wenzhou sobemo-like virus 4* (6/8, 75%), the other four insect-specific viruses are detected in 7 out of 8 (87.5%) mosquito pools with high relative abundance.

### Phylogenetic analysis of reads mapping to pathogens and potential pathogens

**Figure 5.**
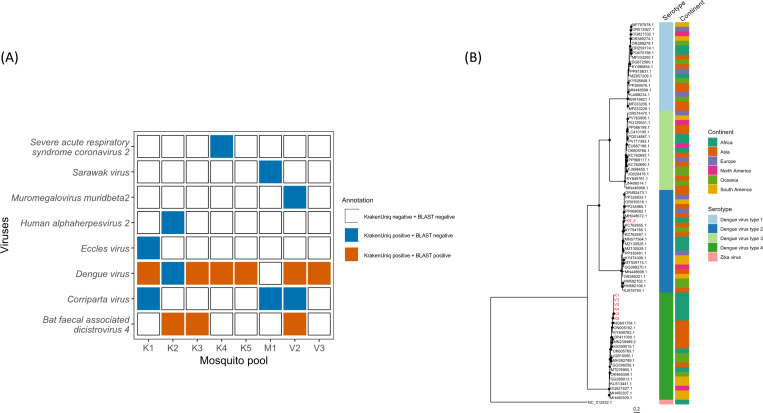
Known human or animal viruses detected in mosquito pools, and phylogenetic analysis of detected DENV RNA. **A)** Annotation results based on KrakenUniq with default nt database and BLAST are shown in different colors. Vermillion rectangulars signify confirmation by two methods used. **B)** Phylogenetic analysis of dengue reads from field mosquito pools. Maximum likelihood phylogenetic trees comparing DENV short reads (~ 100bp) from DRC mosquito pools (red) to reference sequences (black). The tree is constructed using RAxML-NG under the GTR+F+I+G4 model, with NC_012532.1 (Zika virus sequence) as the outgroup. Black dots indicate nodes with bootstrap support greater than 70% across 1,000 replicates.

**Figure 6.**
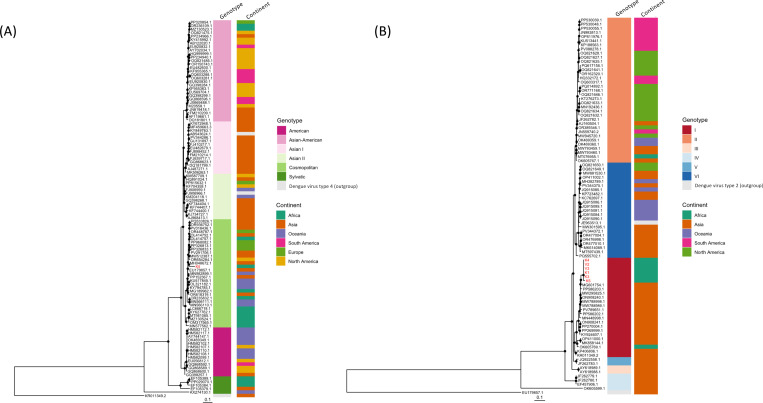
Phylogenetic analysis of DENV-2 and DENV-4 reads from field mosquito pools. Maximum likelihood phylogenetic trees comparing DENV reads from DRC mosquito pools (red) to reference sequences (black), constructed using RAxML-NG (GTR+F+I+G4 model) with KR011349.2 and EU179857.1 as outgroups, respectively. Analysis of the **A)** DENV-2 showing clustering of reads from a single DRC pool (mapping to non-coding region, ~100bp) with DENV-2 reference sequences (>10,000bp), though with limited bootstrap support. and **B)** DENV-4 showing clustering of reads from six DRC pools (mapping to non-coding region, ~100bp) with DENV-4 references sequences (>10,000bp). Black dots indicate nodes with bootstrap support greater than 70% based on 1,000 replicates.

BLAST-confirmed reads mapping to viral species related to human or animal diseases are detected in multiple mosquito pools, including DENV and *bat faecal associated dicistrovirus 4* (Figure 5a). Though SPAdes does not successfully assemble any contigs spanning multiple DENV reads in this study, BLAST-confirmed, merged paired-end reads mapping to DENV are found in 6 out of the 8 (75%) field mosquito pools (Table S7). Reads mapping to DENV-2 in one mosquito pool (K5) are highly similar to one sequence from Malaysia (MH048672.1 10664–10770, 107 bp, nucleotide identity = 94%, Figure 5b and 6a). Reads mapping to DENV-4 found in all six positive pools are short (< 100bp) and show high similarity to the 5’-untranslated (UTR) region of a published sequence from Thailand (MG601754.1 1–90, nucleotide identity = 97.8%) (Figure 5b and 6b). Amplification of polyprotein genes and other fragments using published assays failed ^28–30^, likely due to low DENV RNA concentration and RNA degradation within mosquito pools (median peak RNA fragment length 176 nt, IQR 115–178). We note that these DENV-4 reads also map to Wenzhou sobemo-like virus 4 sequences, but with lower similarity (nucleotide identity ≤ 96.67%) and higher e-values. Phylogenetic analysis of these short, merged paired-end reads suggests the presence of DENV types 2 and 4 in the DRC, though resolution is limited.

**Figure 7.**
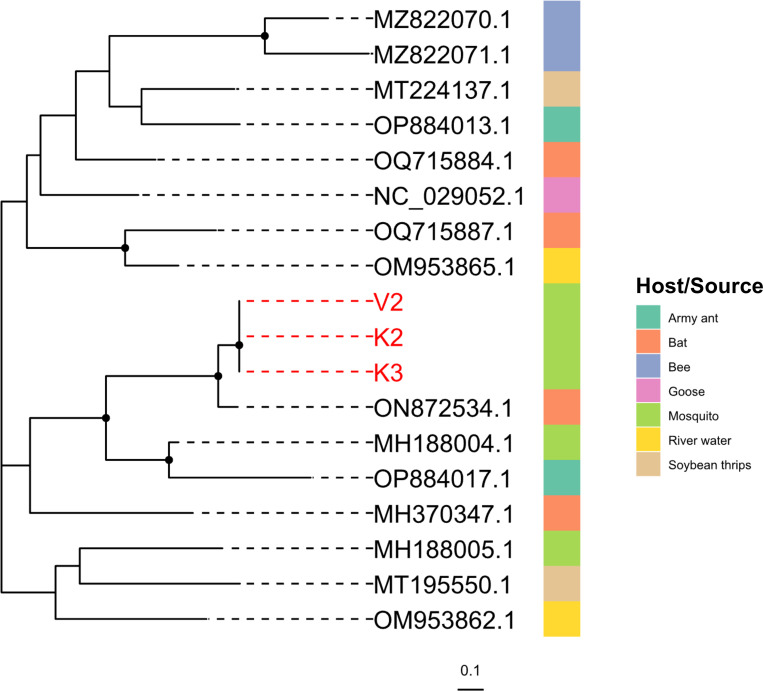
**Phylogenetic analysis of bat faecal associated dicistrovirus 4** reads in DRC field mosquito pools (red) mapping to the non-coding region (7078 – 7330 nt based on ON872534.1) compared to published sequences (black), constructed using RAxML-NG with the HKY+F+G4 model. Black dots indicate nodes with bootstrap support greater than 70% across 1,000 replicates.

We find high similarity between reads mapping to *bat fecal-associated dicistrovirus 4* to a published *bat faecal associated dicistrovirus 4* sequence detected in feces from *Pteropus poliocephalus* from Australia (ON872534.1, 7078–7330, 253 bp) in three mosquito pools (Figure 5a). However, we are unable to assemble contigs for this virus, and resolution of phylogenetic analysis is limited. Additional details on these viral reads are provided in Figure 7 and Table S8.

### PCR confirmation of DENV and mosquito blood meal investigation

Real-time PCR targeting the DENV polyprotein gene, distinct from genomic regions included in our phylogenetic analysis, confirms DENV RNA in the three mosquito pools with highest DENV read counts in metagenomic analysis (Table S7), indicating consistency between next-generation sequencing and real-time PCR within its limits of detection.

**Figure 8.**
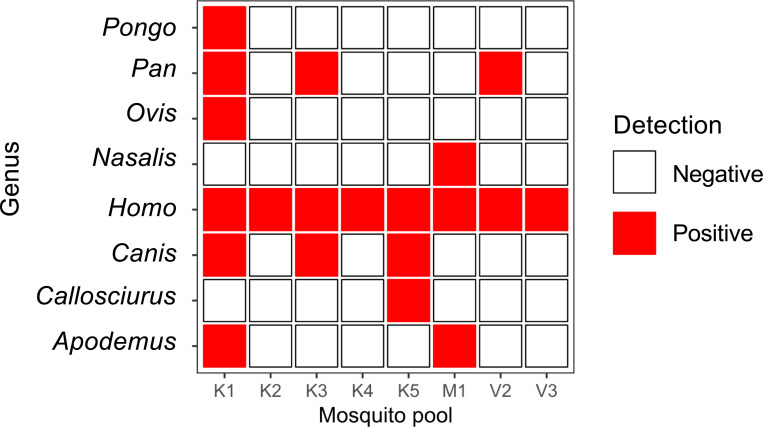
**Mosquito blood meal sources** determined by amplicon sequencing of the 16S ribosomal RNA and cytochrome b genes.

Investigation of mosquito blood meals using amplicon sequencing showed that mosquitoes from all selected pools had taken blood from *Homo sapiens* (Figure 8). Additionally, we detect reads mapping to non-human primates (4/8 pools), rodents (3/8 pools), canines (3/8 pools), and sheep (1/8 pools).

## Discussion

Our findings confirm the presence of dengue and other viruses with human or animal pathogenic potential in wild-caught *Aedes* mosquitoes circulating near the DRC’s border with Angola. The presence of human blood in all mosquito pools suggests active dengue transmission in the Kimpese region, including peri-urban and rural zones outside of the city. The combination of a diverse mosquito virome and the presence of blood meals from multiple mammals suggests robust opportunities for arthropod-borne viral transmission between species, as well as for spillover events. Together, our experience suggests a future role for xenosurveillance and metagenomic sequencing as a complement to existing data systems developed to monitor for emerging infectious diseases in countries like the DRC.

Multiple arboviral disease outbreaks have been documented in the DRC during the last century, including yellow fever, dengue, chikungunya, and others ^3,17,18,21^. Except for DENV, we did not find other known human pathogenic arboviruses in the selected *Aedes* pools. Detection of DENV in mosquito pools by RT-PCR suggests active DENV circulation in the Kimpese region, and metagenomic sequencing reads from small regions of the genome point towards the circulation of both DENV-2 and DENV-4. Three of the four DENV serotypes ^31^ have been recorded in the human population of the DRC (DENV-1, DENV-2, and DENV-3), with DENV-1 being the most frequently reported ^3^. One study in Singapore reported that the majority of the dengue hemorrhagic fever cases involving DENV-2 were from the cosmopolitan genotype ^32^. DENV-2 detected in our study is most similar to the cosmopolitan genotype of DENV-2, which has been previously reported in the DRC ^33^. The DENV-2 reads detected in our study show closer similarity to cosmopolitan DENV-2 from Malaysia than to DENV-2 sequences previously reported from the DRC, however this comparison is based on small genome fragments. Although the DENV-4 reads detected in our study also map to Wenzhou sobemo-like virus 4, an insect-specific virus, they have lower similarity (nucleotide identity ≤96.67%) and higher e-values. Our study provides preliminary evidence that DENV-4 may circulate in *Aedes* mosquitoes from the DRC and therefore infect people in this region.

Data from all *Aedes* mosquito pools in our study confirm human blood meals. Previous studies have reported high dietary diversity of *Aedes* mosquitoes, with a predominant preference for mammalian hosts ^34,35^. We corroborate these findings, with diverse mammalian blood meals observed across mosquito pools, including blood from non-human primates. Non-human primates can be amplifying hosts and play considerable roles in the sylvatic cycle of DENV transmission, which can spark DENV epidemics in nearby human populations ^36^. Detection of rodent, canine, and sheep genomes in the collected mosquito pools illustrate the diversity of hosts of *Aedes*, though further research is needed to confirm the roles of these hosts in DENV transmission, including the contribution to human infection. Notably, three (K2, K4, and V3) mosquito pools appear to have exclusively fed on human blood.

Similar windows of detection have been observed for pathogen RNA and human DNA in mosquitoes following a blood meal. Past work has shown complete digestion of a human blood meal varies depending on the mosquito species and typically takes about 36 hours to 3 days ^37,38^. The persistence of pathogen RNA or DNA in mosquitoes can vary depending on the pathogen type, viral strain, viral load, and mosquito species. Non-arboviral RNA can remain detectable in the mosquito midgut for a field-relevant time-frame—up to 18 hours for west nile virus RNA and up to 24 hours for HIV-1 RNA after blood feeding ^39^. Influenza virus RNA is detectable up to 36 hours post feeding ^38^. For arboviruses, the detection window may be longer. For example, one study showed a reduction in DENV-2 RNA signal as early as 12 hours post-feeding, with little to no signal detected after 24 hours. However, if the virus successfully replicated in mosquito tissues, a subsequent increase in RNA levels was observed between 100 and 200 hours post-blood meal ^38^. The co-detection of human DNA and DENV RNA in the same mosquito pools suggests a common source, likely a viremic human host. Although detection of DENV RNA does not necessarily indicate the presence of infectious virions, its presence in two of these pools (K4 and V3) strongly suggests active DENV circulation in these communities.

In addition to DENV, we identify reads mapping to *bat faecal associated dicistrovirus 4*. Detection of this non-arbovirus might be related to incomplete degradation of the viral material by the mosquito digestive system at the time of sampling. This is the first report of *bat faecal associated dicistrovirus 4* in the DRC. While dicistrovirus is believed to exclusively infect invertebrates, it has been recently reported in blood samples from febrile patients in Tanzania and Nigeria ^40,41^, the intestinal content of a captive red squirrel with enteritis ^42^, and has been identified in free-living gorillas in neighboring Republic of Congo ^43^. Interestingly, we found *bat faecal associated dicistrovirus 4* reads in a mosquito pool (K2) that exclusively fed on human blood, suggesting that it came from an infected human. However, the host range and pathogenicity of these viruses are unclear.

Our findings also shed light on circulating *Aedes* species along the DRC-Angola border. Kimpese comprises a small city and surrounding region where more than 100,000 people are thought to live ^44^, though the DRC has not performed a formal census since the 1950s. It is located in the Kongo Central province, has a tropical climate, and spans mountains, valleys, and grasslands. *Aedes aegypti* and *Ae. albopictus* are the main vectors of several major pathogenic arboviruses, with *Ae. aegypti* being more competent for DENV and ZIKV transmission ^45^. *Ae. aegypti* was previously the dominant *Aedes* species in the DRC, until recent, rapid replacement of native *Aedes* species by *Ae. albopictus* in the western and northern regions of DRC ^15^. Our findings corroborate these changes in the DRC *Aedes* population; most collected *Aedes sp.* mosquitoes are *Ae. albopictus* (98.3%). The highly diverse viromes in our *Aedes* mosquito pools include viruses specific to invertebrates, vertebrates, plants, bacteria, fungi, and protozoa. These reads likely reflect the mosquitoes’ ingestion of blood, nectar, and environment substrates ^46^. However, the large number of unclassified reads within all pools confirms how much remains to be learned about this important mosquito population.

Clustering observed among the samples from Kimpese city and Viaza in PCA provides evidence of geographical differences in local *Aedes* mosquito populations. Most of the top viral genera contributing to the variance in principal components 1 and 2 have been detected in insects, plants, and bacteria. These findings suggest that local ecological and environmental factors—such as elevation, land use patterns, or vegetation—may be shaping the mosquito virome. In addition, the variability observed between mosquito pools within Kimpese city may reflect microenvironmental differences or heterogeneous exposure to viral sources within an urban landscape.

Several limitations of our study should be highlighted. First, mosquitoes were not pooled based on morphological mosquito species. Second, our metagenomic analysis focuses on *Aedes*. Analysis of other mosquito genera would provide broader insights into differences in arboviral transmission and viromes in the DRC. Third, downsampling was necessary to accommodate sequencing costs. We sequence a small number of mosquito pools from sites within one region, which provides useful insights into arboviral transmission in Kimpese but limits the generalizability of our findings to other regions. Fourth, despite positive RT-PCR results and the identification of reads mapping to limited regions of the DENV genome, our attempts to amplify additional and longer DENV fragments using several published assays were unsuccessful, likely due to low RNA concentration and RNA degradation within the mosquito. As a result, we did not have sufficient genomic coverage to construct whole genomes. Nor can metagenomic sequencing tell us whether infectious virions are present or definitively determine dengue types in circulation. Fifth, the use of mosquito pools improved the yield of our sequencing efforts but prevents us from estimating the prevalence of viruses in individual mosquitoes. Finally, viral metagenomic techniques have high sensitivity but low specificity. To overcome this shortcoming, we employ a conservative approach leveraging multiple bioinformatic tools complemented by real-time PCR, both of which confirm the presence of DENV in our sample set.

In conclusion, our study provides new insights into viruses infecting *Aedes* in the DRC and demonstrates the potential of wild-caught mosquito xenosurveillance. We use virome and blood-meal analysis to improve understanding of *Aedes* mosquito feeding behavior in the DRC, and present evidence of endemic DENV transmission in the Kimpese region near the DRC’s border with Angola. These findings are particularly relevant to ongoing discussions about where to offer newly approved dengue vaccines and indicate a need for larger human studies to define dengue epidemiology in the DRC and across Africa ^47^. They also suggest a role for sustained vector control efforts targeting *Aedes* in the DRC, extending beyond outbreak response, and highlight opportunities for future arthropod-borne disease studies in the region.

## Methods

### Mosquito collection

Adult *Aedes* mosquitoes were collected from three health areas in Kimpese, DRC, Ceco (Kimpese city), Malanga, and Viaza (Figure 2a), using electric aspirators (Prokopack Aspirator, John W. Hock, Gainesville, USA) and BG sentinel traps (Biogents Inc, Regensburg, Germany) baited with dry ice. Mosquitoes were collected from April 23 – 30, 2022, during the rainy season when mosquito density is high. All collections were performed outdoors, and GPS coordinates along with a brief description of collection sites were recorded.

Collected mosquitoes were morphologically identified to the species level following the Huang morphological key ^48^. *Aedes* species were pooled by sample collection site. All field mosquito pools were preserved in DNA/RNA Shield (ZYMO research, CA, USA) and stored at −80°C.

Uninfected *Aedes aegypti* mosquitoes (NR-48920) were obtained from the NIH/NIAID Filariasis Research Reagent Resource Center through BEI Resources. A random subset of 20 mosquitoes was pooled to serve as the laboratory uninfected *Ae. aegypti* control.

### Nucleic acid extraction

Eight field *Aedes* mosquito pools, comprising a total of 155 mosquitoes, were randomly selected from the three health areas: five from Kimpese city, one from Malanga, and two from Viaza (Figure 2b). These pools represent approximately 20% of the total mosquito pools from each region. The selected mosquito pools were washed with 70% ethanol and rinsed twice with molecular-grade water before removal of mosquito heads ^49^. Total RNA was extracted from mosquito bodies from the selected field mosquito pools, the laboratory un-infected *Ae. aegypti* mosquito pool from BEI Resources, and one water control using the Quick-RNA Tissue/Insect kit (ZYMO research, CA, USA) following the manufacturer’s instructions. DNase I treatment using DNase I Set (ZYMO research, CA, USA) was carried out during the RNA extraction process.

### Mosquito species confirmation and blood meal investigation

To confirm the mosquito species, two regions within the cytochrome c oxidase I (*COI*), and one region within the second internal transcribed spacer (*ITS2*) genes were amplified from RNA extracted from the mosquito pools using published primers and conditions as shown in Table S9 ^50–52^. DNA-based methods have been widely used in mosquito blood meal analysis and provide reliable results ^53^. The 16S ribosomal RNA (*16S*) and cytochrome *b* (*cytB*) genes are commonly used due to their species-specific resolution ^54,55^. In this study, we amplified *16S* and *cytB* from RNA extracted from field-collected mosquito pools using published primers (Table S9) ^56,57^. All amplicons from the same mosquito pool were combined in equimolar amounts. Then, 68 ng of DNA from each pooled amplicon was used for library construction. Amplicon libraries were constructed using the Native Barcoding Kit 96 V14 and sequenced on a Flongle Flow Cell (R10.4.1) using the MinION platform (Oxford Nanopore Technologies, Oxford, United Kingdom).

### Ribosomal RNA depletion, library preparation, and viral metagenomic sequencing

To increase viral metagenomic sequencing efficiency, mosquito ribosomal RNA (rRNA) depletion was carried out before library preparation using the NEBNext RNA Depletion Core Reagent set (New England BioLabs, MA, USA) with published probes targeting *Aedes* mosquitoes ^58^. RNA fragment lengths after rRNA depletion were assessed for five pools using Agilent TapeStation 4150 with High Sensitivity RNA ScreenTape (Agilent, Waldbronn, Germany). Sequencing libraries were prepared and barcoded using the NEBNext Ultra II Directional RNA Library Prep Kit and the Multiplex Oligos for Illumina (96 Unique Dual Index Primer Pairs) kit (New England BioLabs, Massachusetts, USA), respectively. The barcoded libraries were sequenced using the NovaSeq S4 platform (Illumina, California, USA) at the High-Throughput Sequencing Facility (HTSF) at the University of North Carolina at Chapel Hill to generate 150 bp paired-end reads. The expected total yield was 4–5 billion reads.

### Dengue virus confirmation by real-time PCR

To confirm DENV calls made by sequencing, we tested RNA from all mosquito pools using a published real-time PCR assay to detect pan-DENV RNA with the SuperScript III Platinum One-Step qRT-PCR kit (Life Technologies, Carlsbad, CA, USA) ^59^.

### Data analysis

To investigate viruses in the mosquito pools, Illumina NovaSeq sequencing reads were demultiplexed into individual samples based on their barcode sequences. Paired-end reads were then merged into single reads based on overlap detection using BBMerge (version 38.96) ^60^, followed by adapter removal using Trimmomatic (version 0.36) with default parameters, and filtering short reads by setting the minimum length as 36 ^61^. Reads mapping to human (GRCh38.p14), *Ae. albopictus* (Aalbo_primary.1), and *Ae. aegypti* (AaegL5.0) genomes, as well as the *Ae. simpsoni* CO1 gene (KT881399.1) were removed using bwa-mem2 (version 2.2.1) ^62^ and SAMtools (version 1.21) ^63^.

Reads remaining in the laboratory un-infected *Ae. aegypti* mosquito pool and water control pool were assembled into contigs using metaSPAdes (version 4.0.0) with default parameters ^64^. These contigs were considered as contaminating contigs. Reads in field mosquito pools were aligned to the contaminating contigs using bwa-mem2 (version 2.2.1), and mapping reads were removed using SAMtools (version 1.21). Taxonomic classification of the remaining reads were performed using KrakenUniq (version 1.0.4) in default mode, with the standard NCBI-nt database downloaded on July 31, 2023 ^65^. Then, reads from field mosquitoes mapping to sequences annotated as viral genera present in the laboratory uninfected *Ae. aegypti* mosquito pool and water control were excluded from the analysis. A viral genus or family was considered present by KrakenUniq if it had at least 10 mapped reads per million viral reads based on the KrakenUniq reports using the default NCBI-nt database ^26^. If the total read count of a specific viral genus or family in a specific library is less than 0.1% of the highest read count for that viral genus or family within the same sequencing lane, then it was considered as a false-positive due to index-hopping ^66^. The relative abundance of viral genera and families was calculated based on the KrakenUniq reports with default NCBI-nt database. This was done by dividing the number of reads mapping to specific taxonomic groups by the total number of viral reads, then multiplying the result by 100. Reads classified as viruses were extracted for further analysis. Principal component analysis (PCA) was performed based on the viral genus annotation.

BLAST (version 2.14.1), an alignment-based method, was used to confirm the classification of reads mapping to human and animal pathogens, as well as potential pathogens. BLAST-confirmed reads mapping to human and animal pathogens or potential pathogens were assembled into contigs using metaSPAdes (version 4.0.0) with default parameters. The longest contigs or consensus sequences generated from merged paired-end reads for pathogens and potential pathogens in the detected pools were selected and aligned with representative reference sequences (Table S10-S15) from NCBI Virus, RefSeq, as well as GenBank databases, including diverse genotypes/serotypes spanning multiple geographic regions and collection years, using MAFFT (version 7.490) with default parameters. Phylogenetic trees were generated using the maximum likelihood (ML) method in RAxML-NG (version 1.2.2) ^67^, with the best-fit available model selected based on the Bayesian Information Criterion (BIC) values in IQ-TREE (version 2.4.0) ^68^.

For Nanopore sequencing data used for mosquito species determination and blood meal analysis, raw data in Fast5 file format from the sequencer were used for base-calling with Guppy (version 5.6.7), with a phred quality score ≥ 6 (Q score ≥ 6). Reads were demultiplexed according to their barcodes, which were then trimmed using Guppy with defaults. BLAST was applied to all passed reads to investigate the mosquito species and blood sources (calls were made for hits with e-value < 1e-6 and length > 100bp).

All data was visualized in R software (version 4.2.0; R Core Team, Vienna, Austria) in RStudio (version 2022.02.2) with *ggrepel* (version 0.9.6), *ggplot2* (version 3.5.2), *tidyverse* (version 2.0.0), *eulerr* (version 7.0.2), *viridis* (version 0.6.5), *scico* (version 1.5.0), *ape* (version 5.8–1), *ggtree* (version 3.16.0), *rentrez* (version 1.2.4), *countrycode* (version 1.6.1), *RColorBrewer* (version 1.1–3), and *dplyr* (version 1.1.4) packages. Mapping was done using *sf* (version 1.0.21), *rnaturalearth* (version 1.0.1), and *rnaturalearthdata* (version 1.0.0) packages in R software. Data for mapping was downloaded from the Global Administrative Areas Database (GADM) ^69^ and the Humanitarian Data Exchange (HDX) ^70^.

## Supplementary Material

Supplement 1

Supplement 2

Supplement 3

## Data Availability

NovaSeq and MinION sequencing data presented in the study are deposited in the NCBI Sequence Read Archive (BioProject ID: PRJNA1200724 and PRJNA1200731).
