# Supplementary material for: Evidence of dengue virus transmission and a diverse Aedes mosquito virome on the Democratic Republic of Congo-Angola border
Source: bioRxiv. 2025 Sep 23:2025.01.16.633031. Preprint. [Version 2] doi: 10.1101/2025.01.16.633031 (PMC12485970; doi:10.1101/2025.01.16.633031)
Supplement: Supplement 1 [file media-1.pdf]

Table S1. Mosquito species identified by morphology at each collection site

Location	*Aedes albopictus*	*Aedes aegypti*	*Aedes simpsoni*	Total
Kimpese city	239	1	2	242
Malanga	312	3	1	316
Viaza	102	4	0	106
Total	653	8	3	664

Table S2. Mosquito species identification using Nanopore sequencing data

Sample name	Absolute number of classified reads mapping to *Aedes albopictus*	Absolute number of classified reads mapping to *Aedes aegypti*	Absolute number of classified reads mapping to *Aedes simpsoni*
K1	114	3	0
K2	412	8	0
K3	167	5	0
K4	145	5	0
K5	172	5	0
M1	43	6	0
V2	151	3	0
V3	175	1	0

Table S3. Genera in the laboratory un-infected *Ae. aegypti* mosquito pool and water control identified using the krakenUniq default nt database (de novo assembled contigs, generated from raw reads after human and mosquito filtering)

Laboratory mosquito pool	Water control
Chryseobacterium	*Acinetobacter*
Pseudomonas	*Cutibacterium*
Enterobacter	*Herbaspirillum*
Penicillium	*Methylophilus*
Cupriavidus	*Corynebacterium*
Aspergillus	*Gemella*
Azospirillum	*Hathewaya*
Staphylococcus	*Malassezia*
Leucobacter	*Ralstonia*
Wallemia	*Delftia*
Acinetobacter	*Inhella*
Variovorax	*Photorhabdus*
Comamonas	*Sphingomonas*
Sphingomonas	*Nocardioides*
Dyadobacter	*Mycobacterium*
Marinilongibacter	*Mycolicibacillus*
Siphonobacter	*Kocuria*
Pedobacter	*Rothia*
Gordonia	*Modestobacter*
Rhodococcus	*Kutzneria*
Betanodavirus	*Staphylococcus*
	Streptococcus
	Veillonella
	Peptoniphilus
	Cloacibacterium
	Babesia
	Natronomonas

Table S4. Viral genera identified in laboratory un-infected *Ae. aegypti* mosquito pool and water control using KrakenUniq with default nt database (reads could not be *de novo* assembled to contigs)

Laboratory mosquito pool	Water control
Quaranjavirus	*Pahexavirus*
Betanodavirus	*Elvirus*
	Sextaecvirus
	Andhravirus
	Schiekvirus
	Samunavirus
	Haloferacalesvirus
	Vojvodinavirus
	Sanovirus
	Gammaretrovirus
	Picobirnavirus
	Betacoronavirus
	Allexivirus
	Parapoxvirus
	Mimivirus
	Inovirus
	Betapapillomavirus

Table S5. Top ten viral genera contributing to principal components 1 (PC1) and 2 (PC2) during PCA

PC1	PC2
Certrevirus	*Orthophasmavirus*
Elunavirus	*Orthoflavivirus*
Ghunavirus	*Cripavirus*
Jacunavirus	*Mimivirus*
Mitovirus	*Simplexvirus*
Namakavirus	*Tombusvirus*
Narnavirus	*Ourmiavirus*
Roskildevirus	*Alphamesonivirus*
Phasivirus	*Jonvirus*
Iflavirus	*Alphabaculovirus*

Table S6. Read counts of the most prevalent insect-specific viruses (reads/million reads mapping to viruses)

	K1	K2	K3	K4	K5	M1	V2	V3
Aedes flavivirus	1,320	58,252	656	268	230	0	284	71
Wenzhou sobemo-like virus 4	246,875	0	314,077	356,127	53,343	0	211,721	320,021
Hubei mosquito virus 2	330	0	228	278	76	604,697	337,739	9,509
Guangzhou sobemo-like virus	160,691	1,618	148,055	96,652	30,291	0	103,348	145,950
Sichuan mosquito sobemo-like virus	98,890	1,618	51,417	83,487	21,927	0	39,073	74,002

Table S7. DENV read counts, reads per million viral reads identified using the KrakenUniq nt database, and multiplex real-time PCR results using the pan-DENV assay published by Waggoner *et al.* (*Emerg Infect Dis*. 2016)

Sample	DENV reads, n	DENV reads/million viral reads	Coordinates [reference]	DENV real-time PCR
K1	1	10.3	1-90[1]	Negative
K2	0	0	0	Negative
K3	14	31.9	1-90[1]	**Positive**
K4	83	68.7	1-91[1]	**Positive**
K5	65	27.7	1-91[1], 10664-10770[2]	Negative
M1	0	0	0	Negative
V2	28	17.3	1-91[1]	Negative
V3	114	32.1	1-91[1]	**Positive**

[1] *Based on published DENV-4 sequence: MG601754.1*
[2] *Based on published DENV-2 sequence: MH048672.1*

Table S8. Read counts of *bat faecal associated dicistrovirus 4*

Sample	*Bat faecal associated dicistrovirus 4* reads, n	Coordinates[1]	Total viral reads (based on nt database)	*Bat faecal associated dicistrovirus 4* reads/million viral reads, n (based on nt database)
K1	0	0	96956	0
K2	33	7078-7330	2472	13349.5
K3	182	7078-7330	439000	414.6
K4	0	0	1207424	0
K5	0	0	2204216	0
M1	1	0	1316904	0
V2	52	7078-7330	1618920	32.1
V3	0	0	3555364	0

[1] *Coordinates are based on published Bat faecal associated dicistrovirus 4 sequence: ON872534.1*

Table S9. Primers for mosquito species determination and blood meal investigation. Reaction mixtures and reaction conditions are the same for all assays.

Target	Primer sequences 5′ to 3′	Reaction mixture	Reaction Condition	Study
16S	CGGTTGGGGTGACCTCGGA<br>GCTGTTATCCCTAGGGTAACT	5 µl 5× PrimeSTAR GXL Buffer (Takara Bio)<br>1 µl PrimeSTAR GXL DNA Polymerase (Takara Bio)<br>4 µl dNTP Mixture (2.5 mM each)<br>200 nM Primer 1<br>200 nM Primer 2<br>2 µl Template<br>Add H_2O to 50 µl	35 cycles of:<br>98°C for 10 s<br>55°C for 15 s<br>68°C for 1 min;<br>Final extension at 68°C for 2 min;<br>Hold at 4°C	Taylor, 1996
cytB	CCATCCAACATCTCAGCATGATGAAA<br>GCCCCTCAGAATGATATTTGTCCTCA			Meyer, Hofelein, Luthy, & Candrian, 1995
COX1	TACAGTTGGAATAGACGTTGATAC<br>TCCAATGCACTAATCTGCCATATTA			Zhang & Hewitt, 1996
COI	GGTCAACAAATCATAAAGATATTGG<br>TAAACTTCAGGGTGACCAAAAAATCA			Folmer, 1994
ITS2	GCTCGTGGATCGATGAAGAC<br>TGCTTAAATTTAGGGGGTGTAGTCAC			Batovska, Blacket, Brown, & Lynch, 2016

Table S10. Sequences included in dengue virus phylogenetic analysis (all types)

Accession number/Sample name	Virus name	Length, nt	Geographical origin	Continent	This study
NC012532.1	Zika virus	10794	Uganda	Africa	no
OR259174.1	Dengue virus type 1	10641	Nigeria: Oyo	Africa	no
PQ470156.1	Dengue virus type 1	10660	Ghana: Accra	Africa	no
MZ857206.1	Dengue virus type 1	10179	Kenya	Africa	no
MN577564.1	Dengue virus type 2	10276	Kenya: Wajir	Africa	no
MZ130525.1	Dengue virus type 2	10597	Cameroon	Africa	no
MZ130528.1	Dengue virus type 2	10597	Djibouti	Africa	no
OK605766.1	Dengue virus type 3	10707	Somalia	Africa	no
PV717393.1	Dengue virus type 3	10560	Kenya: Mombasa	Africa	no
PQ014887.1	Dengue virus type 3	10360	Ethiopia: Afar	Africa	no
OK605769.1	Dengue virus type 4	10651	Senegal	Africa	no
MT076955.1	Dengue virus type 4	10648	Kenya: Kisumu	Africa	no
MH450307.1	Dengue virus type 4	10649	Venezuela: Aragua	South America	no
MH450309.1	Dengue virus type 4	10649	Venezuela: Aragua	South America	no
GQ398270.1	Dengue virus type 2	10724	Puerto Rico	North America	no
KY474308.1	Dengue virus type 2	10648	Ecuador: Machala	South America	no
OQ821332.1	Dengue virus type 1	10598	Costa Rica	North America	no
OR389274.1	Dengue virus type 1	10600	Brazil	South America	no
MF797878.1	Dengue virus type 1	10687	Ecuador	South America	no
OR389321.1	Dengue virus type 2	10512	Peru	South America	no
OR039518.1	Dengue virus type 2	10528	Brazil: Santa Catarina, Sao Jose	South America	no
MT929715.1	Dengue virus type 2	10046	Brazil: Uberaba	South America	no
PQ129531.1	Dengue virus type 3	10680	USA: California	North America	no
EU687198.1	Dengue virus type 3	10675	USA: Puerto Rico	North America	no
PV763906.1	Dengue virus type 3	10592	Brazil: Sao Paulo, Sao Paulo	South America	no
OQ821627.1	Dengue virus type 4	10511	Cuba	North America	no
KU513441.1	Dengue virus type 4	10650	Brazil: Cambe, PR	South America	no
GU289913.1	Dengue virus type 4	10649	Colombia	South America	no
KX059015.1	Dengue virus type 4	10263	Sri Lanka	Asia	no
MF033200.1	Dengue virus type 1	10714	Singapore	Asia	no
MF033228.1	Dengue virus type 1	10735	Singapore	Asia	no
MF033206.1	Dengue virus type 1	10735	Singapore	Asia	no
KC762655.1	Dengue virus type 2	10723	Indonesia: Makassar	Asia	no
MN448698.1	Dengue virus type 2	10464	Thailand	Asia	no
KC762657.1	Dengue virus type 2	10723	Indonesia: Makassar	Asia	no
KY849761.1	Dengue virus type 3	10660	Laos: Vientiane	Asia	no

KC762692.1	Dengue virus type 3	10707	Indonesia: Makassar	Asia	no
KC762690.1	Dengue virus type 3	10709	Indonesia: Makassar	Asia	no
KY849762.1	Dengue virus type 4	10603	Laos: Salavan	Asia	no
GQ398256.1	Dengue virus type 4	10653	Singapore	Asia	no
PP269576.1	Dengue virus type 1	10622	Viet Nam	Asia	no
MN448598.1	Dengue virus type 1	10179	Thailand	Asia	no
KY496854.1	Dengue virus type 1	10689	Taiwan	Asia	no
PP234965.1	Dengue virus type 2	10667	Sri Lanka	Asia	no
OR492473.1	Dengue virus type 2	10685	India	Asia	no
KJ918750.1	Dengue virus type 2	10723	India	Asia	no
LC410195.1	Dengue virus type 3	10667	Thailand	Asia	no
MN448968.1	Dengue virus type 3	10663	Thailand	Asia	no
PP586198.1	Dengue virus type 3	10700	Thailand	Asia	no
OP411000.1	Dengue virus type 4	10648	Singapore	Asia	no
ON005192.1	Dengue virus type 4	10550	Thailand	Asia	no
MN239489.2	Dengue virus type 4	10509	India	Asia	no
KJ468234.1	Dengue virus type 1	10622	Germany	Europe	no
OR512927.1	Dengue virus type 1	10672	Italy	Europe	no
PP815631.1	Dengue virus type 1	10027	Spain: Granada	Europe	no
PP326833.1	Dengue virus type 2	10664	France	Europe	no
PP968082.1	Dengue virus type 2	10704	Russia: Novosibirsk	Europe	no
PP335481.1	Dengue virus type 2	10176	France	Europe	no
OR574470.1	Dengue virus type 3	10642	Italy	Europe	no
PP968117.1	Dengue virus type 3	10707	Russia: Novosibirsk	Europe	no
KR919821.1	Dengue virus type 1	10735	Australia	Oceania	no
DQ672560.1	Dengue virus type 1	10735	French Polynesia	Oceania	no
KY926848.1	Dengue virus type 1	10597	French Polynesia: Faa'a commune	Oceania	no
OR389279.1	Dengue virus type 1	10601	French Polynesia	Oceania	no
KY794785.1	Dengue virus type 2	10657	Papua New Guinea: Lihir	Oceania	no
HM582102.1	Dengue virus type 2	10713	New Caledonia	Oceania	no
HM582108.1	Dengue virus type 2	10713	French Polynesia: Tahiti	Oceania	no
FJ898455.1	Dengue virus type 3	10663	Cook Islands	Oceania	no
JQ920476.1	Dengue virus type 3	10671	French Polynesia: Tahiti	Oceania	no
JN406514.1	Dengue virus type 3	10707	Australia	Oceania	no
JQ915090.1	Dengue virus type 4	10246	Wallis and Futuna	Oceania	no
MH382789.1	Dengue virus type 4	10653	Australia: Queensland	Oceania	no
OK469359.1	Dengue virus type 4	10485	Kiribati	Oceania	no
MG601754.1	Dengue virus type 4	10772	Thailand	Asia	no
K1	Dengue virus type 4	90	Democratic Republic of the Congo	Africa	yes
K3	Dengue virus type 4	90	Democratic Republic of the Congo	Africa	yes
K4	Dengue virus type 4	90	Democratic Republic of the Congo	Africa	yes
K5	Dengue virus type 4	90	Democratic Republic of the Congo	Africa	yes
V2	Dengue virus type 4	90	Democratic Republic of the Congo	Africa	yes
V3	Dengue virus type 4	90	Democratic Republic of the Congo	Africa	yes

MH048672.1	Dengue virus type 2	10785	Malaysia	Asia	no
K5_2	Dengue virus type 2	107	Democratic Republic of the Congo	Africa	yes

Table S11. Sequences included in dengue virus type 2 phylogenetic analysis

Accession number/ Sample name	Virus name	Genotype	Length, nt	Geographical origin	Continent	This study
OR654284.1	Dengue virus type 2	Cosmopolitan	10563	USA	North America	no
EU179857.1	Dengue virus type 2	Cosmopolitan	10709	Brunei	Asia	no
EU482679.1	Dengue virus type 2	Asian I	10678	Vietnam	Asia	no
FJ898452.1	Dengue virus type 2	Asian I	10679	Thailand	Asia	no
OL414757.1	Dengue virus type 2	Cosmopolitan	10711	Cambodia	Africa	no
KF955363.1	Dengue virus type 2	Asian-American	10531	Puerto Rico	North America	no
HQ999999.1	Dengue virus type 2	Asian-American	10725	Guatemala: Guatemala City	North America	no
GQ868592.1	Dengue virus type 2	American	10667	Colombia	South America	no
KR011349.2	Dengue virus type 4	Cosmopolitan	10644	Philippines	Asia	no
K5	Dengue virus type 2	Cosmopolitan	107	Democratic Republic of the Congo	Africa	yes
MH048672.1	Dengue virus type 2	Cosmopolitan	10785	Malaysia	Asia	no
OR236109.1	Dengue virus type 2	Asian-American	10507	Reunion	Africa	no
KY627762.1	Dengue virus type 2	Cosmopolitan	10675	Burkina Faso	Africa	no
OR235832.1	Dengue virus type 2	Cosmopolitan	10507	Reunion	Africa	no
MG189962.1	Dengue virus type 2	Cosmopolitan	10665	Tanzania	Africa	no
MZ130524.1	Dengue virus type 2	Cosmopolitan	10597	Benin	Africa	no
MT981085.1	Dengue virus type 2	Cosmopolitan	10668	Mauritania	Africa	no
MN577562.1	Dengue virus type 2	Cosmopolitan	10716	Kenya: Mombasa	Africa	no
LC666718.1	Dengue virus type 2	Cosmopolitan	10724	Ghana: Accra	Africa	no
OM317565.1	Dengue virus type 2	Cosmopolitan	10630	Cameroon: Africa	Africa	no
MZ130523.1	Dengue virus type 2	Asian-American	10597	Cameroon	Africa	no
PP029070.1	Dengue virus type 2	Sylvatic	10090	Senegal	Africa	no
OQ821485.1	Dengue virus type 2	Asian-American	10512	Mexico	North America	no
KF955365.1	Dengue virus type 2	Asian-American	10780	Venezuela	South America	no
PP234940.1	Dengue virus type 2	Asian-American	10608	Honduras	North America	no
AY702034.1	Dengue virus type 2	Asian-American	10722	Cuba	North America	no
KY415992.1	Dengue virus type 2	Asian-American	10724	Haiti	North America	no
AB122020.1	Dengue virus type 2	Asian-American	10723	Dominican Republic	North America	no
KM587709.1	Dengue virus type 2	Asian II	10713	USA	North America	no
OR150743.1	Dengue virus type 2	Asian-American	10609	USA: FL	North America	no
EU920832.1	Dengue virus type 2	Asian-American	10698	French Guiana	South America	no
OQ821475.1	Dengue virus type 2	Asian-American	10512	Haiti	North America	no
EU920830.1	Dengue virus type 2	Asian-American	10717	French Guiana	South America	no
M20558.1	Dengue virus type 2	Asian-American	10723	Jamaica	North America	no
OQ603288.1	Dengue virus type 2	Asian-American	10725	Colombia: Huila	South America	no

OQ603281.1	Dengue virus type 2	Asian-American	10725	Colombia: Huila	South America	no
MK506263.1	Dengue virus type 2	Asian I	10688	Thailand	Asia	no
OL414752.1	Dengue virus type 2	Cosmopolitan	10714	Cambodia	Asia	no
PV018436.1	Dengue virus type 2	Cosmopolitan	10723	Singapore	Asia	no
AJ968413.1	Dengue virus type 2	Asian II	10723	Taiwan:Hsiao-Liu-Chiu Islet, Ping Tong	Asia	no
PP152367.1	Dengue virus type 2	Cosmopolitan	10720	Nepal: Kathmandu	Asia	no
PV344286.1	Dengue virus type 2	Asian I	10652	Thailand	Asia	no
MF459663.3	Dengue virus type 2	Asian I	10724	China	Asia	no
PQ533826.1	Dengue virus type 2	Cosmopolitan	10680	Pakistan: Balochistan	Asia	no
PV291706.1	Dengue virus type 2	Cosmopolitan	10599	Thailand	Asia	no
PP234966.1	Dengue virus type 2	Asian-American	10573	Indonesia	Asia	no
OR936752.1	Dengue virus type 2	Cosmopolitan	10642	Pakistan	Asia	no
OR618319.1	Dengue virus type 2	Cosmopolitan	10638	Taiwan	Asia	no
PP320854.1	Dengue virus type 2	Asian-American	10668	France	Europe	no
PP968082.1	Dengue virus type 2	Cosmopolitan	10704	Russia: Novosibirsk	Europe	no
OR448787.1	Dengue virus type 2	Cosmopolitan	10663	Switzerland	Europe	no
PP326833.1	Dengue virus type 2	Cosmopolitan	10664	France	Europe	no
PP326813.1	Dengue virus type 2	Cosmopolitan	10723	France	Europe	no
PP815632.1	Dengue virus type 2	Asian II	10010	Spain: Granada	Europe	no
MN982899.1	Dengue virus type 2	Cosmopolitan	10720	Australia: Rockhampton	Oceania	no
KU517845.1	Dengue virus type 2	Cosmopolitan	10723	Papua New Guinea	Oceania	no
HM582108.1	Dengue virus type 2	American	10713	French Polynesia: Tahiti	Oceania	no
HM582099.1	Dengue virus type 2	American	10714	Fiji	Oceania	no
AY744147.1	Dengue virus type 2	American	10713	Tonga	Oceania	no
KX274130.1	Dengue virus type 2	Sylvatic	10736	Australia	Oceania	no
HM582112.1	Dengue virus type 2	American	10714	Tonga	Oceania	no
HM582102.1	Dengue virus type 2	American	10713	New Caledonia	Oceania	no
OL321182.1	Dengue virus type 2	Cosmopolitan	10635	Papua New Guinea: NCD, Hohola	Oceania	no
MN566110.1	Dengue virus type 2	Cosmopolitan	10704	New Caledonia	Oceania	no
MN566111.1	Dengue virus type 2	Cosmopolitan	10722	New Caledonia	Oceania	no
KY794785.1	Dengue virus type 2	Cosmopolitan	10657	Papua New Guinea: Lihir	Oceania	no
KM204118.1	Dengue virus type 2	Asian II	10723	Papua New Guinea	Oceania	no
OK469349.1	Dengue virus type 2	American	10591	Samoa	Oceania	no
FJ906966.1	Dengue virus type 2	Asian II	10678			no
AB543624.1	Dengue virus type 2	Asian I	10731			no
MW512387.1	Dengue virus type 2	Cosmopolitan	10238	Singapore	Asia	no
JN819418.1	Dengue virus type 2	Asian-American	10576	Viet Nam	Asia	no
KY849763.1	Dengue virus type 2	Asian I	10669	Laos	Asia	no
GU131897.1	Dengue virus type 2	Asian I	10428	Cambodia	Asia	no
FM210214.1	Dengue virus type 2	Asian I	10685	Vietnam	Asia	no
FJ410217.1	Dengue virus type 2	Asian I	10678	Vietnam	Asia	no
GQ868623.1	Dengue virus type 2	Asian I	10677	Cambodia	Asia	no
FJ639717.1	Dengue virus type 2	Asian I	10678	Cambodia	Asia	no

KY672948.1	Dengue virus type 2	Asian I	10723	China	Asia	no
DQ181799.1	Dengue virus type 2	Asian I	10723	Thailand	Asia	no
AJ487271.1	Dengue virus type 2	Asian I	10597	Thailand	Asia	no
KF744407.1	Dengue virus type 2	Asian II	10176	Philippines	Asia	no
KF744404.1	Dengue virus type 2	Asian II	10176	Philippines	Asia	no
KF744400.1	Dengue virus type 2	Asian II	10176	Philippines	Asia	no
KJ734727.1	Dengue virus type 2	Asian II	10723	Taiwan	Asia	no
KF704358.1	Dengue virus type 2	Asian II	10176	Cuba	North America	no
HQ891024.1	Dengue virus type 2	Asian II	10615	Taiwai	Asia	no
FJ906959.1	Dengue virus type 2	Asian II	10678	Papua	Oceania	no
GQ398268.1	Dengue virus type 2	Asian II	10724	Indonesia	Asia	no
JX669488.1	Dengue virus type 2	Asian-American	10724	Brazil	South America	no
GQ868596.1	Dengue virus type 2	Asian-American	10667	Venezuela	South America	no
EU569704.1	Dengue virus type 2	Asian-American	10668	Puerto Rico	North America	no
GQ398299.1	Dengue virus type 2	Asian-American	10723	Puerto Rico	North America	no
EU482600.1	Dengue virus type 2	Asian-American	10679	Nicaragua	North America	no
GQ398284.1	Dengue virus type 2	Asian-American	10723	Puerto Rico	North America	no
AF119661.1	Dengue virus type 2	Asian-American	10723	China	Asia	no
FM210209.1	Dengue virus type 2	Asian-American	10692	Vietnam	Asia	no
DQ181801.1	Dengue virus type 2	Asian-American	10723	Thailand	Asia	no
GQ868589.1	Dengue virus type 2	American	10668	Mexico	North America	no
GQ868600.1	Dengue virus type 2	American	10636	Puerto Rico	North America	no
EU056812.1	Dengue virus type 2	American	10714	Puerto Rico	North America	no
HM582110.1	Dengue virus type 2	American	10713	FrenchPolynesia	Oceania	no
HM582117.1	Dengue virus type 2	American	10714	Tonga	Oceania	no
HM582107.1	Dengue virus type 2	American	10713	USA	North America	no
GQ398257.1	Dengue virus type 2	American	10713	Indonesia	Asia	no
EF105389.1	Dengue virus type 2	Sylvatic	10723	Senegal	Africa	no
EF105384.1	Dengue virus type 2	Sylvatic	10724	Senegal	Africa	no
EF105379.1	Dengue virus type 2	Sylvatic	10719	Malaysia	Asia	no

Table S12. Sequences for dengue virus type 4 phylogenetic analysis

Accession number/ Sample name	Virus name	Genotype	Length, nt	Geographical location	Continent	This study
KR011349.2	Dengue virus type 4	I	10664	Philippines	Asia	no
EU179857.1	Dengue virus type 2		10709	Brunei	Asia	no
KY924607.1	Dengue virus type 4	I	10161	Viet Nam	Asia	no
MW793460.1	Dengue virus type 4	II	10648	Thailand	Asia	no
AY618989.1	Dengue virus type 4	III	10653	Thailand	Asia	no
EF457906.1	Dengue virus type 4	IV	10666	Malaysia	Asia	no
JF262780.1	Dengue virus type 4	IV	10667	Malaysia	Asia	no
JF262783.1	Dengue virus type 4	V	10659	India	Asia	no
JQ915089.1	Dengue virus type 4	VI	10572	New Caledonia	Oceania	no
JQ922558.1	Dengue virus type 4	V	10626	India	Asia	no
KC762697.1	Dengue virus type 4	VI	10653	Indonesia	Asia	no
K1	Dengue virus type 4	I	90	Democratic Republic of the Congo	Africa	yes
K3	Dengue virus type 4	I	90	Democratic Republic of the Congo	Africa	yes
K4	Dengue virus type 4	I	90	Democratic Republic of the Congo	Africa	yes
K5	Dengue virus type 4	I	90	Democratic Republic of the Congo	Africa	yes
V2	Dengue virus type 4	I	90	Democratic Republic of the Congo	Africa	yes
V3	Dengue virus type 4	I	90	Democratic Republic of the Congo	Africa	yes
MG601754.1	Dengue virus type 4	I	10772	China	Asia	no
MT076955.1	Dengue virus type 4	II	10648	Kenya: Kisumu	Africa	no
OK605769.1	Dengue virus type 4	I	10651	Senegal	Africa	no
KT276273.1	Dengue virus type 4	II	10649	Haiti	North America	no
OQ821634.1	Dengue virus type 4	II	10511	Dominican Republic	North America	no
OQ821632.1	Dengue virus type 4	II	10511	Puerto Rico	North America	no
OQ821628.1	Dengue virus type 4	II	10376	Cuba	North America	no
OQ821625.1	Dengue virus type 4	II	10511	Cuba	North America	no
PP530048.1	Dengue virus type 4	II	10164	Brazil	South America	no
HQ332172.1	Dengue virus type 4	II	10649	Venezuela	South America	no
PP530039.1	Dengue virus type 4	II	10164	Brazil	South America	no
OQ603317.1	Dengue virus type 4	II	10100	Colombia: Quindio	South America	no
MW945720.1	Dengue virus type 4	II	10167	Dominica	North America	no
OQ821641.1	Dengue virus type 4	II	10099	Cuba	North America	no
OQ821646.1	Dengue virus type 4	II	10376	El Salvador	North America	no
KP188563.1	Dengue virus type 4	II	10654	Brazil	South America	no
PQ617158.1	Dengue virus type 4	II	10604	USA: FL	North America	no
OP811976.1	Dengue virus type 4	II	10164	Paraguay	South America	no
KU513441.1	Dengue virus type 4	II	10650	Brazil: Cambe, PR	South America	no

PV088278.1	Dengue virus type 4	II	10291	Colombia: Cundinamarca, Girardot	South America	no
JF262782.1	Dengue virus type 4	II	10649	Haiti	North America	no
OR162320.1	Dengue virus type 4	II	10508	USA: FL	North America	no
PQ014892.1	Dengue virus type 4	II	10229	Panama: Cocle	North America	no
OR771168.1	Dengue virus type 4	II	10512	USA: FL	North America	no
OQ821633.1	Dengue virus type 4	II	10511	Haiti	North America	no
OR389346.1	Dengue virus type 4	II	10511	El Salvador	North America	no
MN192436.1	Dengue virus type 4	II	10650	USA	North America	no
OQ821627.1	Dengue virus type 4	II	10511	Cuba	North America	no
JN983813.1	Dengue virus type 4	II	10649	Brazil: Boa Vista, Roraima State	South America	no
PP530055.1	Dengue virus type 4	II	10164	Brazil	South America	no
OQ821649.1	Dengue virus type 4	VI	10511	Jamaica	North America	no
OQ821650.1	Dengue virus type 4	VI	10376	Dominica	North America	no
JN559740.2	Dengue virus type 4	II	10604	Brazil: Boa Vista-RR	South America	no
KP723482.1	Dengue virus type 4	VI	10653	China: Guangdong	Asia	no
OP411000.1	Dengue virus type 4	I	10648	Singapore	Asia	no
MW788989.1	Dengue virus type 4	I	10607	Myanmar: Yangon	Asia	no
MW295825.1	Dengue virus type 4	I	10426	China: Guangzhou	Asia	no
OR477010.1	Dengue virus type 4	VI	10304	Philippines	Asia	no
PP586202.1	Dengue virus type 4	I	10632	Thailand	Asia	no
ON908241.1	Dengue virus type 4	I	10584	Cambodia	Asia	no
PP586203.1	Dengue virus type 4	I	10651	Thailand	Asia	no
MW881530.1	Dengue virus type 4	VI	10319	China: Guangzhou	Asia	no
MN448998.1	Dengue virus type 4	I	10597	Thailand	Asia	no
OR477004.1	Dengue virus type 4	VI	10526	Philippines	Asia	no
MW793459.1	Dengue virus type 4	II	10648	Thailand	Asia	no
MW788998.1	Dengue virus type 4	I	10605	Myanmar: Yangon	Asia	no
AY618988.1	Dengue virus type 4	III	10653	Thailand: Bangkok	Asia	no
MK858144.1	Dengue virus type 4	I	10503	India	Asia	no
KJ160504.1	Dengue virus type 4	II	10650	Sri Lanka	Asia	no
MT597439.1	Dengue virus type 4	VI	10252	South Korea	Asia	no
PQ555702.1	Dengue virus type 4	VI	10649	Malaysia: Negeri Sembilan	Asia	no
MK614089.1	Dengue virus type 4	VI	10350	China: Guangzhou	Asia	no
PV344375.1	Dengue virus type 4	VI	10652	Thailand	Asia	no
PP269999.1	Dengue virus type 4	I	10536	Viet Nam	Asia	no
OP411002.1	Dengue virus type 4	VI	10653	Singapore	Asia	no
PV789651.1	Dengue virus type 4	I	10662	China: Yunnan,Xishuangbanna	Asia	no
OR476998.1	Dengue virus type 4	VI	10526	Philippines	Asia	no
PV344372.1	Dengue virus type 4	VI	10617	Thailand	Asia	no
KP406806.1	Dengue virus type 4	I	10664	South Korea	Asia	no

MW301595.1	Dengue virus type 4	VI	10281	China: Guangzhou	Asia	no
OK605767.1	Dengue virus type 4	II	10648	Indonesia	Asia	no
PP270004.1	Dengue virus type 4	I	10536	Viet Nam	Asia	no
ON908240.1	Dengue virus type 4	I	10582	Myanmar	Asia	no
OK469359.1	Dengue virus type 4	II	10485	Kiribati	Oceania	no
JQ915086.1	Dengue virus type 4	VI	10572	New Caledonia: Dumbea	Oceania	no
JQ915084.1	Dengue virus type 4	VI	10573	French Polynesia: Tahiti	Oceania	no
OK469360.1	Dengue virus type 4	II	10579	Niue	Oceania	no
JQ915081.1	Dengue virus type 4	VI	10572	French Polynesia: Taha'a	Oceania	no
JQ915090.1	Dengue virus type 4	VI	10246	Wallis and Futuna	Oceania	no
JQ915085.1	Dengue virus type 4	VI	10572	New Caledonia: Noumea	Oceania	no
MH382789.1	Dengue virus type 4	VI	10653	Australia: Queensland	Oceania	no
JF262779.1	Dengue virus type 4	IV	10667	Malaysia	Asia	no
OK605599.1	Dengue virus type 4	IV	10677	Borneo	Asia	no
JE963513.1	Dengue virus type 4	VI	10652			no

Table S13. Sequences for *Bat faecal associated dicistrovirus 4* phylogenetic analysis

Accession number/ Sample name	Virus name	Host/Source	This study
K2	Bat faecal associated dicistrovirus 4	Mosquito	yes
K3	Bat faecal associated dicistrovirus 4	Mosquito	yes
MH188004.1	Culex dicistrovirus 1	Mosquito	no
MH188005.1	Culex dicistrovirus 2	Mosquito	no
MT195550.1	Soybean thrips dicistrovirus 1	Soybean thrips	no
MZ822070.1	Apis dicistrovirus 2	Bee	no
MZ822071.1	Apis dicistrovirus 3	Bee	no
OM953862.1	Flumine dicistrovirus 2	River water	no
OM953865.1	Flumine dicistrovirus 3	River water	no
ON872534.1	Bat faecal associated dicistrovirus 4	Bat	no
OQ715884.1	Wenzhou bat dicistrovirus 3	Bat	no
OQ715887.1	Wenzhou bat dicistrovirus 6	Bat	no
V2	Bat faecal associated dicistrovirus 4	Mosquito	yes
MH370347.1	Bat dicistrovirus	Bat	no
OP884017.1	Army ant associated dicistrovirus 7	Army ant	no
OP884013.1	Army ant associated dicistrovirus 4	Army ant	no
MT224137.1	Soybean thrips dicistrovirus 2	Soybean thrips	no
NC_029052.1	Goose dicistrovirus	Goose	no

Table S14. Sequences for *Human pegivirus* phylogenetic analysis

Accession number/ Sample name	Virus name	Host	This study
K4	Human pegivirus	Mosquito	yes
MZ099572.1	Human pegivirus	Homo sapiens	no
ON340918.1	Pegivirus C	Homo sapiens	no
MK684252.1	Human pegivirus	Homo sapiens	no
MW526240.1	Human pegivirus	Homo sapiens	no
MZ099567.1	Human pegivirus	Homo sapiens	no
MN551063.1	Human pegivirus	Homo sapiens	no
OR031224.1	Pegivirus C	Homo sapiens	no
MN551064.1	Human pegivirus	Homo sapiens	no
NC_038437.1	Pegivirus I	Bat	no
MW365447.1	Goose pegivirus	Goose	no
KU351669.1	Pegivirus suis	Porcine	no
NC_034442.1	Pegivirus K	Porcine	no
MF459655.1	Porcine pegivirus	Porcine	no
MG874672.1	Porcine pegivirus	Porcine	no
MT276210.1	Pegivirus equi	Equine	no
NC_020902.1	Equine pegivirus 1	Equine	no
KC410872.1	Equine pegivirus 1	Equine	no
NC_038435.1	Pegivirus G	Bat	no
NC_038434.1	Pegivirus F	Bat	no
LC602140.1	Rodent pegivirus	Rodent	no
LC602141.1	Rodent pegivirus	Rodent	no
OP589986.1	Bat pegivirus	Bat	no
AF176573.1	Hepatitis C virus	Homo sapiens	no
OQ832071.1	Hepatitis C virus	Homo sapiens	no

Table S15. Sequences for *Human blood-associated dicistrovirus* phylogenetic analysis

Accession number/ Sample name	Virus name	Host/Source	This study
OM953859.1	Flumine dicistrovirus 1	River water	no
OQ835731.1	Human blood-associated dicistrovirus	Homo sapiens	no
OR031233.1	Human blood-associated dicistrovirus	Homo sapiens	no
MH370347.1	Bat dicistrovirus	Bat	no
NC_035115.1	Apis dicistrovirus	Bee	no
ON872533.1	Bat faecal associated dicistrovirus 1	Bat	no
OP884011.1	Army ant associated dicistrovirus 3	Army ant	no
OP884017.1	Army ant associated dicistrovirus 7	Army ant	no
OQ715887.1	Wenzhou bat dicistrovirus 6	Bat	no
KY354239.1	Apis dicistrovirus	Bee	no
MH188004.1	Culex dicistrovirus 1	Mosquito	no
MH188005.1	Culex dicistrovirus 2	Mosquito	no
MZ822070.1	Apis dicistrovirus 2	Bee	no
MZ822071.1	Apis dicistrovirus 3	Bee	no
MZ822072.1	Apis dicistrovirus 4	Bee	no
NC_031688.1	Mosquito dicistrovirus	Mosquito	no
K4	Human blood-associated dicistrovirus	Mosquito	yes
K5	Human blood-associated dicistrovirus	Mosquito	yes
MT224137.1	Soybean thrips dicistrovirus 2	Soybean thrips	no
OM953862.1	Flumine dicistrovirus 2	River water	no
OM953865.1	Flumine dicistrovirus 3	River water	no
