# Supplementary material for: Evidence of dengue virus transmission and a diverse Aedes mosquito virome on the Democratic Republic of Congo-Angola border
Source: bioRxiv. 2025 Sep 23:2025.01.16.633031. Preprint. [Version 2] doi: 10.1101/2025.01.16.633031 (PMC12485970; doi:10.1101/2025.01.16.633031)
Supplement: Supplement 3 [file NIHPP2025.01.16.633031v2-supplement-3.pdf]

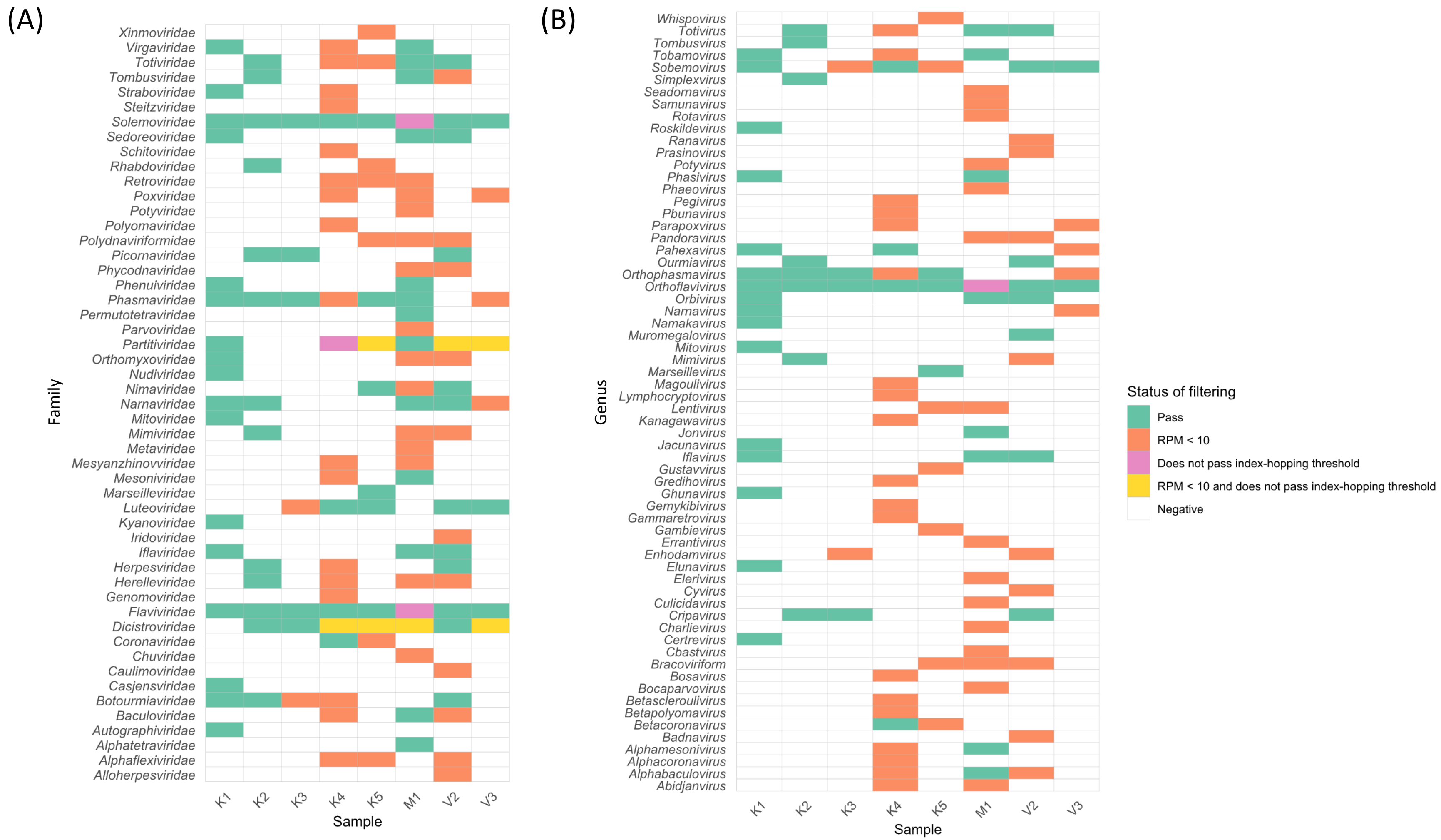

Figure S1. **Filtering results by viral family and genus.** After applying the RPM < 10 threshold and index-hopping filter, **A)** a total of 33 unique total viral families are detected, with each pool containing between 3-17 passed viral families, and **B)** a total of 28 unique total viral genera are detected, with each pool containing between 2-16 passed viral genera. Shaded cells have detected reads, with color denoting filtering result.

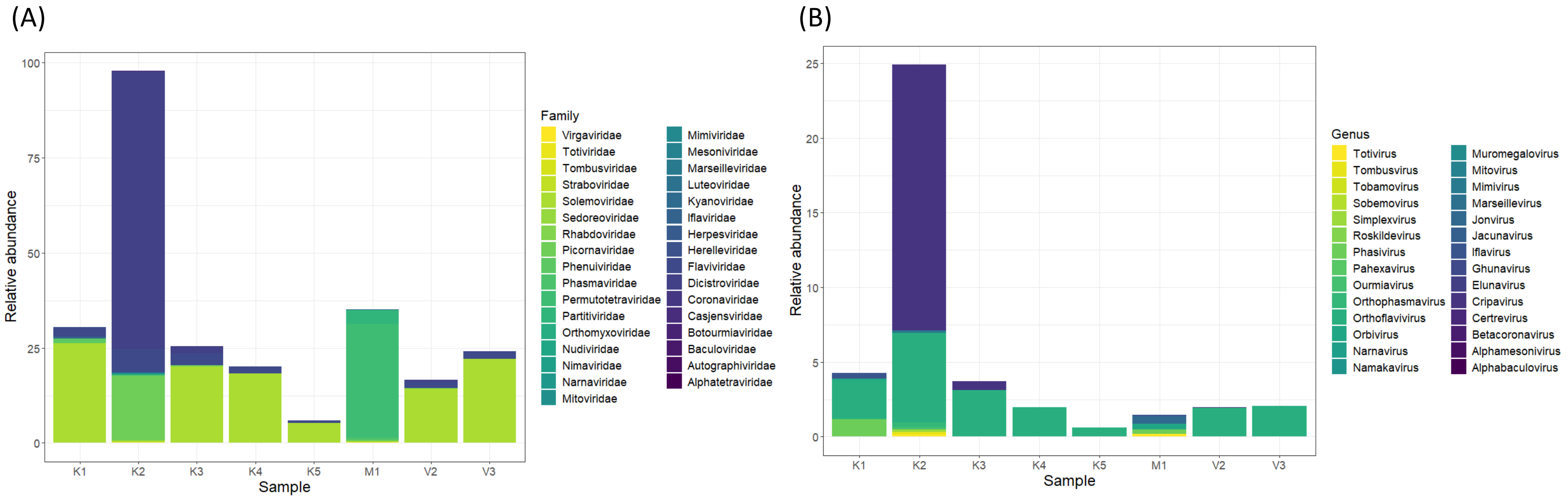

Figure S2. **Viral families and genera detected in field mosquito pools** (after filtering out those with <10 RPM or evidence of index-hopping). **A)** Top five families in each field mosquito pool. **B)** Top five genera in each field mosquito pool. Per International Committee on Taxonomy of Viruses recommendations, relative abundance of *Orthoflavivirus* comprises both *Orthoflavivirus* and *Flavivirus* identified by KrakenUniq.

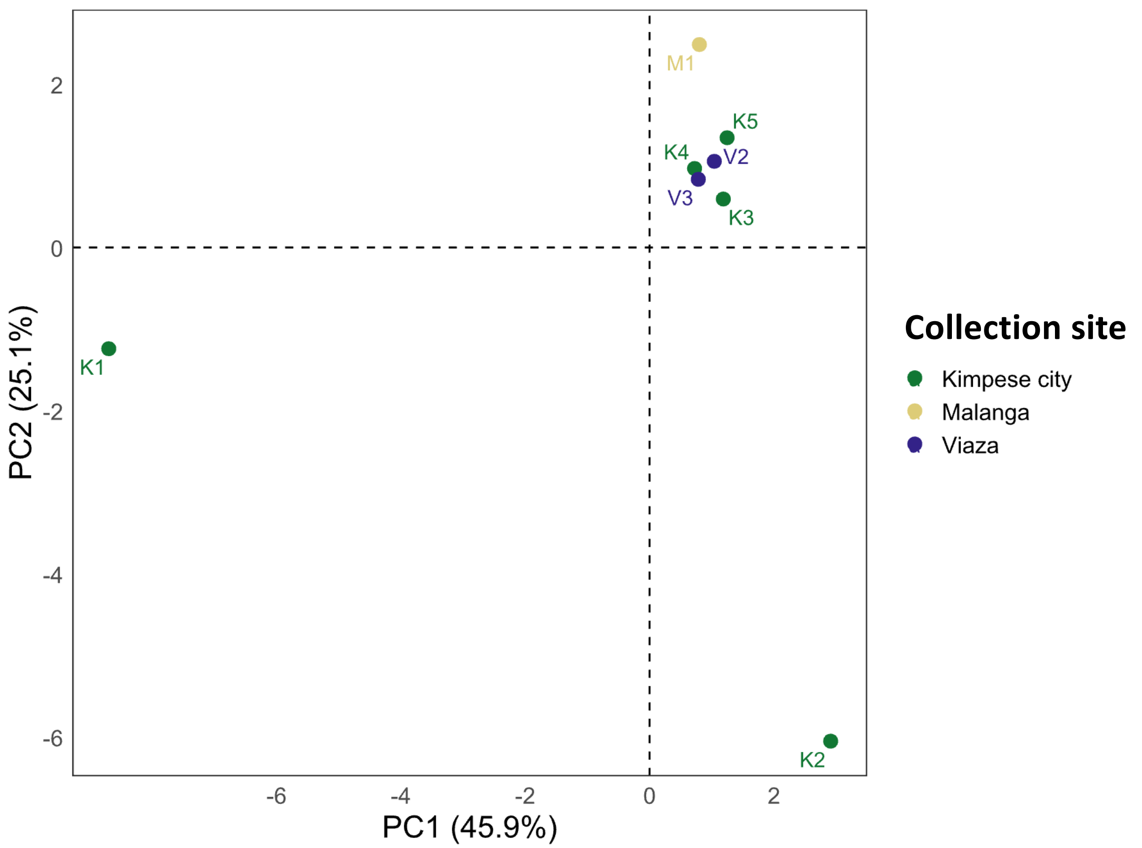

Figure S3. Principal component analysis (PCA) annotated by collection site using a data matrix containing relative abundance of viral genera (after applying the 10 RPM threshold and index-hopping filter).

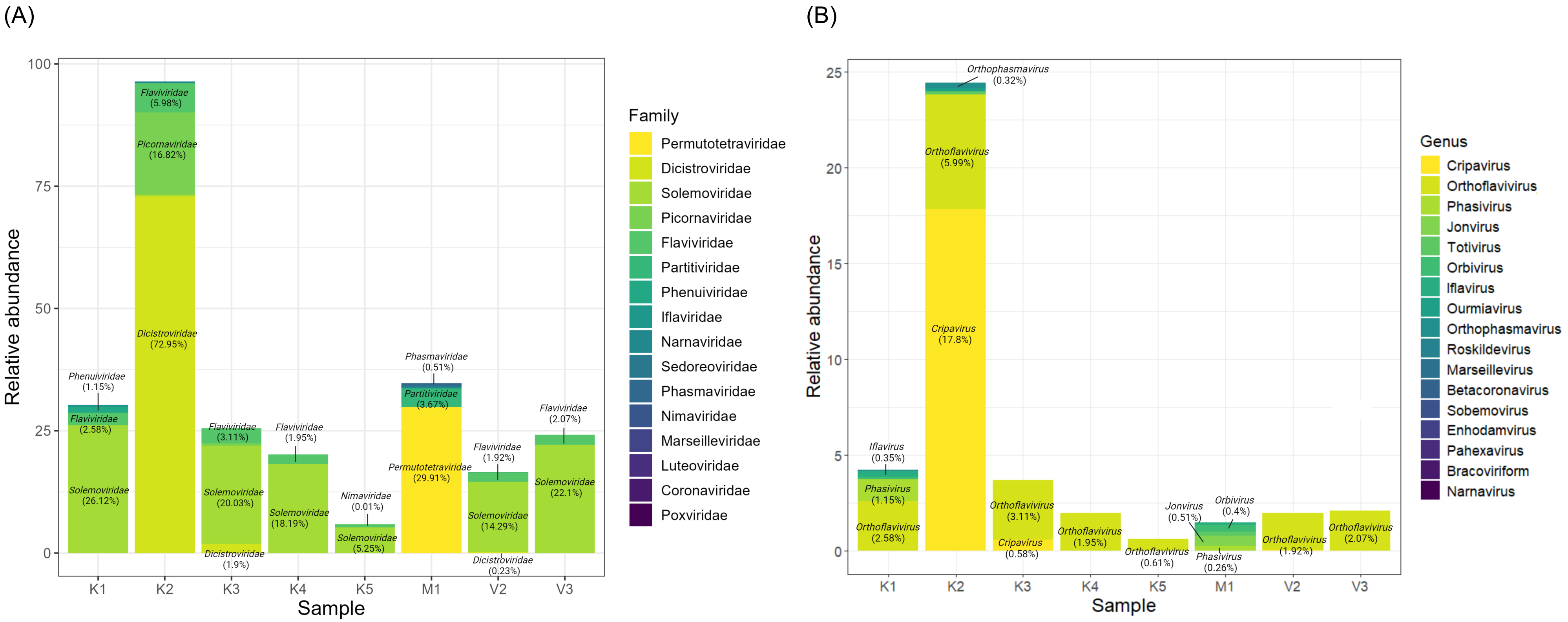

Figure S4. Viral families and genera in field mosquito pools (before filtering out those with <10 RPM or evidence of index-hopping). **A)** Top five families in each field mosquito pool; the top three families with relative abundance >0.1% in each pool are labeled. **B)** Top five genera in field mosquito pools; the top three genus with relative abundance >0.1% in each pool are labeled. Per International Committee on Taxonomy of Viruses recommendations, relative abundance of *Orthoflavivirus* comprises both *Orthoflavivirus* and *Flavivirus* identified by KrakenUniq.

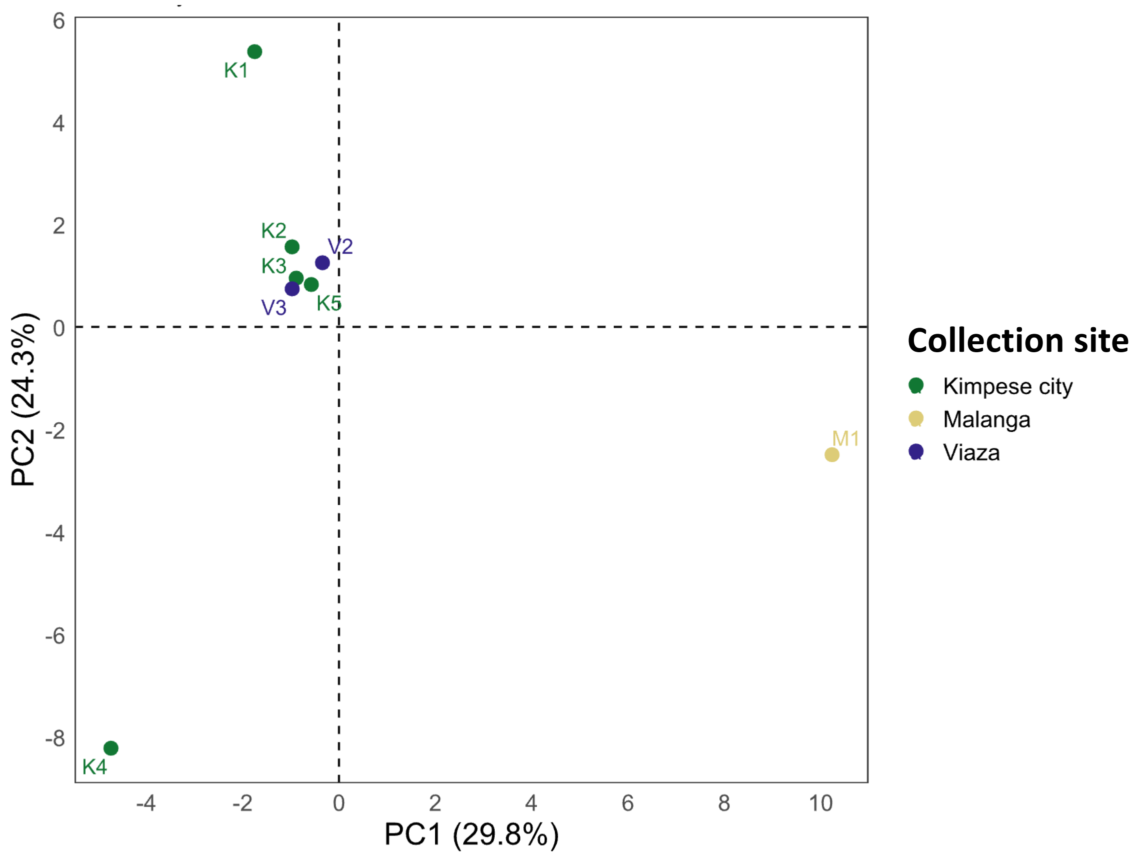

Figure S5. Principal component analysis (PCA) annotated by collection site using a data matrix containing relative abundance of viral genera (before applying the 10 RPM threshold and index-hopping filter).

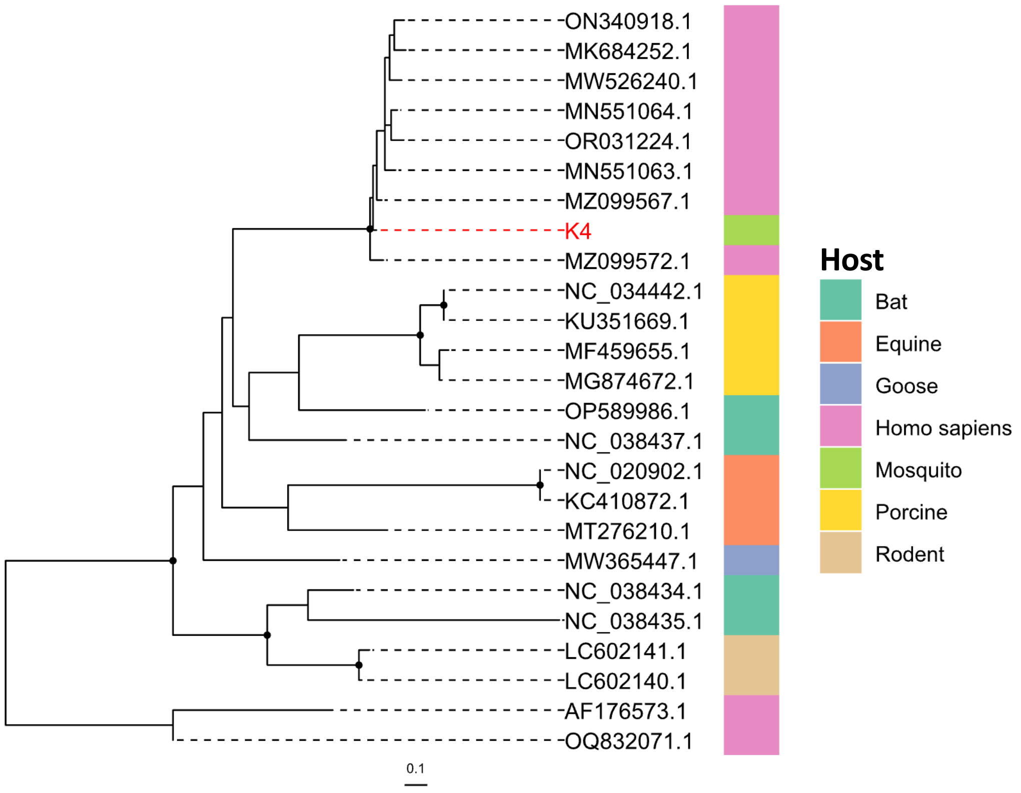

Figure S6. Phylogenetic analysis of pegivirus reads from a DRC field mosquito pool (red) compared to the polyprotein gene region of published sequences (black), constructed using RAxML-NG with the HKY+F+G4 model and hepatitis C virus sequences (AF176573.1 and OQ832071.1) as outgroups. Nodes with >70% bootstrap support across 1,000 replicates are annotated by a black circle.

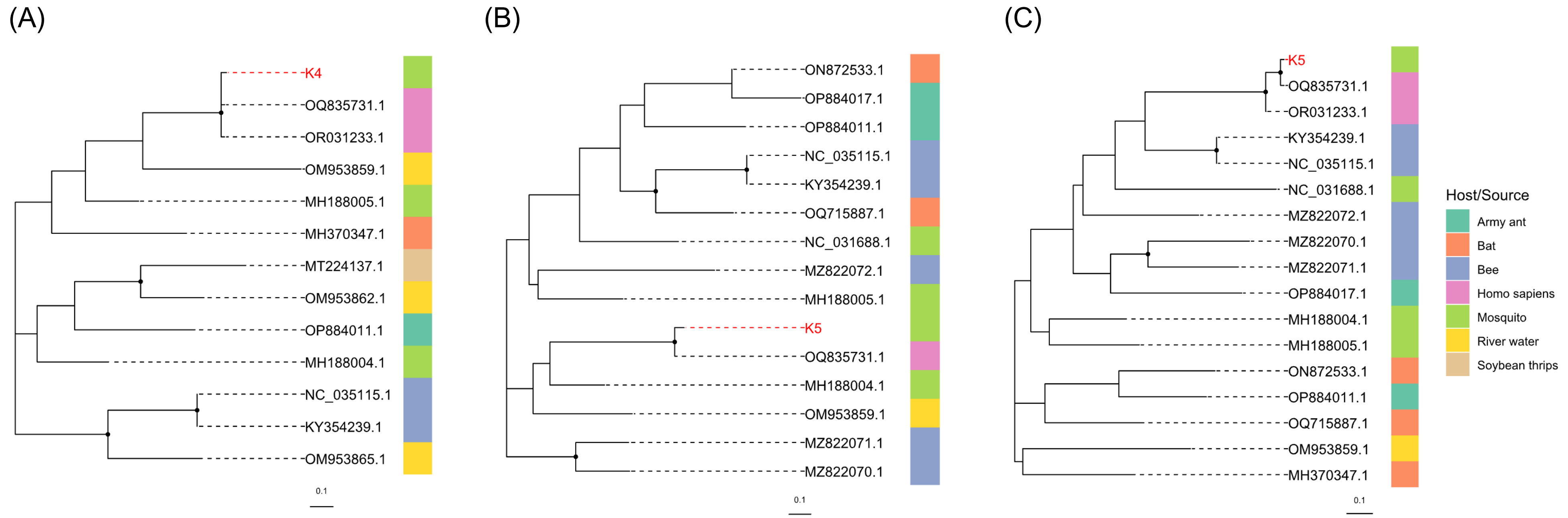

Figure S7. Phylogenetic analysis of human blood-associated dicistrovirus reads in DRC field mosquito pools (red) compared to published sequences (black), constructed using RAxML-NG with the HKY+F+I, HKY+F, and TPM3+F+R2 models, respectively. Analysis of reads from pools **A)** K4 and **B)** K5 mapping to the open reading frame 2 (ORF2) region, and **C)** from K5 mapping to the open reading frame 1 (ORF1) region. Nodes with >70% bootstrap support across 1,000 replicates are annotated by a black circle.
